# A New Bone Substitute Developed from 3D-Prints of Polylactide (PLA) Loaded with Collagen I: An In Vitro Study

**DOI:** 10.3390/ijms18122569

**Published:** 2017-11-29

**Authors:** Ulrike Ritz, Rebekka Gerke, Hermann Götz, Stefan Stein, Pol Maria Rommens

**Affiliations:** 1 Department of Orthopaedics and Traumatology, BiomaTiCS, University Medical Center, Johannes Gutenberg University, 55131 Mainz, Germany; rebekka.gerke@gmx.de (R.G.); pol.rommens@unimedizin-mainz.de (P.M.R.); 2 Platform for Biomaterial Research, University Medical Center, BiomaTiCS, Johannes Gutenberg University, 55131 Mainz, Germany; hgoetz@uni-mainz.de; 3 Georg-Speyer-Haus—Institute for Tumor Biology and Experimental Therapy, 60659 Frankfurt, Germany; s.stein@gsh.uni-frankfurt.de

**Keywords:** 3D-printing, polylactide, collagen, biocompatibility, osteogenesis, angiogenesis

## Abstract

Although a lot of research has been performed, large segmental bone defects caused by trauma, infection, bone tumors or revision surgeries still represent big challenges for trauma surgeons. New and innovative bone substitutes are needed. Three-dimensional (3D) printing is a novel procedure to create 3D porous scaffolds that can be used for bone tissue engineering. In the present study, solid discs as well as porous cage-like 3D prints made of polylactide (PLA) are coated or filled with collagen, respectively, and tested for biocompatibility and endotoxin contamination. Microscopic analyses as well as proliferation assays were performed using various cell types on PLA discs. Stromal-derived factor (SDF-1) release from cages filled with collagen was analyzed and the effect on endothelial cells tested. This study confirms the biocompatibility of PLA and demonstrates an endotoxin contamination clearly below the FDA (Food and Drug Administration) limit. Cells of various cell types (osteoblasts, osteoblast-like cells, fibroblasts and endothelial cells) grow, spread and proliferate on PLA-printed discs. PLA cages loaded with SDF-1 collagen display a steady SDF-1 release, support cell growth of endothelial cells and induce neo-vessel formation. These results demonstrate the potential for PLA scaffolds printed with an inexpensive desktop printer in medical applications, for example, in bone tissue engineering.

## 1. Introduction

During the last decade, much knowledge has been acquired in trauma management. However, large segmental bone defects caused by trauma, infection, bone tumors or revision surgeries still represent a challenge for trauma surgeons all over the world [1,2]. Until now, the gold standard for treatment of large bone defects is autologous bone grafting, which, unfortunately, is associated with severe problems; one or several additional interventions are needed and the transplant material is limited [3]. Although several materials and various implant options have been developed, the perfect solution for filling up critical size defects still remains to be found [4].

New and innovative bone substitutes, which should be non-toxic, endotoxin-free, biocompatible and optimally biodegradable as well as osteoinductive, are needed. One problem concerning bone substitutes is the fact, that they need high mechanical stability and should simultaneously express a porous structure for ingrowth of bone, tissue and cells. Moreover, bone substitutes should stimulate osseointegration. This could be achieved by modifications of the material with active compounds displaying the requested features. One possible solution are materials consisting of a mechanical stable structure (e.g., hydroxyapatite) [5] filled, coated or modified with polymers representing soft part, e.g., hydrogels [6], which can be altered with cells or bioactive molecules [7]. Many studies in the last years demonstrated that the critical point for bone tissue engineering is not the induction of bone growth but the supply of the implant with nutrients and oxygen, showing that vascularization of the implant and its surroundings is the main requirement [8,9]. Therefore, new bone substitutes should also elicit angiogenic effects. One option to create the requested effects together with osteogenesis is the immobilization of bioactive molecules, for example incorporation of SDF-1 (stromal-derived factor), which is known to act chemotactically on endothelial (progenitor) cells and thereby to induce angiogenesis [10,11].

Three-dimensional (3D) printing is a simple procedure to create 3D porous scaffolds used for bone tissue engineering [12]. A promising material is polylactide, a polymer which is known to be degraded by hydrolysis to harmless and non-toxic monomers [13]. Moreover, it displays mechanical stability, which is why it has been studied and employed in various medical studies and applications [14,15].

One soft material being used together with mechanically stable materials is collagen. Collagens are extracellular matrix (ECM) molecules and are widely employed as biomaterials due to their excellent behaviour [16], even in bone tissue engineering [17]. It is inexpensive, well-tolerated and easy to handle and modify. Moreover it is degraded in the body by collagenases, releasing non-toxic and non-immunogenic peptides [18]. Collagens from various sources exist [19], but rat tail or bovine sources are the most commonly used. Collagen has been used for tissue engineering [20,21] and delivery of various bioactive molecules [22] for decades, emphasizing its potential as a biomaterial for various medical applications. Besides bone tissue engineering studies, collagen-based (hydro)gels or scaffolds, have been suggested, inter alia, for use in cartilage repair [23], wound-infection [24], tendon tissue engineering [25], neurodegenerative diseases [26] and nucleus pulposus regeneration [27]. Moreover, Bersini et al. combined computational tools with an experimental approach including hydrogels consisting of fibrin and collagen to engineer vascularized bone-mimicking tissues [28].

In the present study, solid discs as well as porous cage-like 3D structures made of PLA were coated and filled with collagen. These materials were employed for biocompatibility evaluation and testing of endotoxin contamination. The effects on various cell types were tested in vitro and interpreted with regard to their potential usefulness in medical applications.

## 2. Results and Discussion

### 2.1. Scaffold Characterization

**Figure 1:**
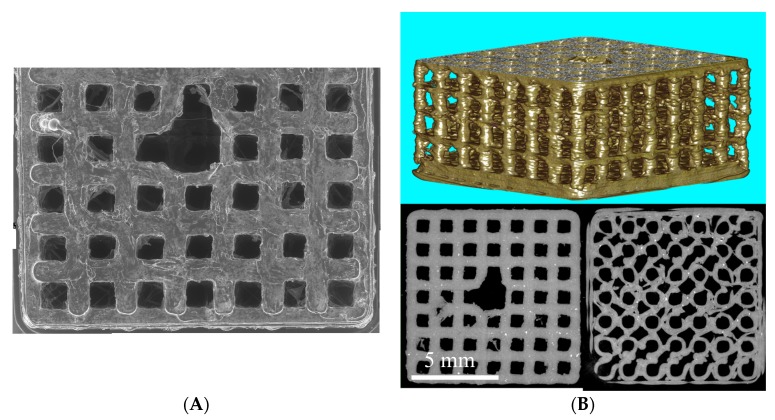
(**A**) Scanning electron microscope (SEM) image gallery of the PLA scaffolds. Single Images (100×) are stacked together manually; (**B**) µCT 3D volume rendering of polylactide (PLA) cage and µCT-slices as multiplanar reconstruction (MPR) images from the top and bottom site of the PLA cage.

PLA scaffolds were fabricated using a 3D-printer, Ultimaker 2+, with a 0.25 mm nozzle. Images taken by scanning electron microscopy shown in Figure 1 demonstrate the rough and inherent surface of the printed 3D models due to the limited resolution of the 3D-printer. The three-dimensional reconstruction of the cage-like model as well as the inside views taken by a microcomputed tomography technique demonstrate a scaffold structure with pores large enough for cells to invade from the top as well as from side walls. The comparison of the designed 3D model and the printed results demonstrates some structural imperfections due to the limiting factors of the 3D print technology. Threads and loose particles as well as geometric deviations caused by the fabrication process are estimated as non-relevant and consciously accepted. The hole in the center of the scaffold was designed to facilitate gel injection into the scaffold.

Polylactide is a promising starting material for scaffolds used for biomedical application. However, not many studies utilized low-cost and “easy-to-handle” desktop printers, which can be afforded and handled by everyone [29,30]. The structure of the printed scaffolds hints to positive properties for cell growth as well as modification options; both facts being prerequisites for a material usable in bone tissue engineering.

### 2.2. Endotoxin Contamination

Endotoxins are lipopolysaccharides (LPS), located on the outer cell membrane of Gram-negative bacteria. Ubiquitously present, they can enter the blood stream of humans and induce immunological responses resulting in inflammation and infection, possibly leading to multiple organ failure and eventually to death [31,32]. Once contaminated, removal of endotoxins from infected materials is almost impossible as they are resistant to high temperature and even acidic solutions. Only extreme high temperatures or extreme acidic solutions can destroy endotoxins—conditions that would destroy most biomaterials. Therefore, avoiding endotoxin contamination during syntheses processes is highly recommended [33]. To our knowledge, no studies have been performed to determine endotoxin contamination in PLA scaffolds after 3D printing.

**Figure 2:**
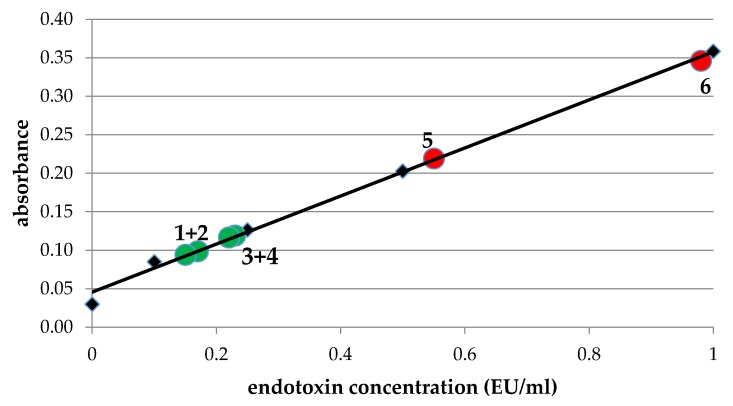
Determination of endotoxin concentration in PLA disks. Endotoxin concentration was determined in supernatants of PLA strings before printing directly after unpacking (red circle 5) and after a few days at the air (red circle 6) and in PLA disks after printing incubated for 24 h (1 + 2) or 48 h (3 + 4): green circles. The trendline corresponds to standards with 0.1, 0.25, 0.5 and 1.0 EU/mL (black quarders). The diagram shows that endotoxin concentrations are well below the FDA limit of 0.5 EU/mL.

In order to ensure, that polylactide scaffolds do not contain endotoxins after printing, we performed limulus amebocyte lysate-assays (LAL-assays) to determine endotoxin contamination before and after printing in the supernatant of PLA incubated in PBS (phosphate buffered saline, Figure 2). PLA was used directly after taking it out of the package (red circle 5) as well as after being in the air for a few days (red circle 6) and analyzed for endotoxin contamination; LAL-assay demonstrates an endotoxin contamination well above the FDA-limit (0.5 EU/mL). In contrast, LAL-assay demonstrated an endotoxin contamination of our PLA printed scaffolds from 0.1–0.25 EU/mL (Figure 2, green circles), which is clearly below the FDA-limit (0.5 EU/mL).

Most likely the high temperatures (up to 240 °C) during printing processes are sufficiently high to destroy any present endotoxins. Similar findings were reported by Neches et al. who describe the intrinsic sterility of 3D printing due to, among other factors, the high fusion modeling temperatures [34]. After printing, the PLA disks or -scaffolds are transferred directly to ethanol-containing solutions and handled under sterile conditions to minimize further endotoxin contaminations.

### 2.3. Biocompatibility

**Figure 3:**
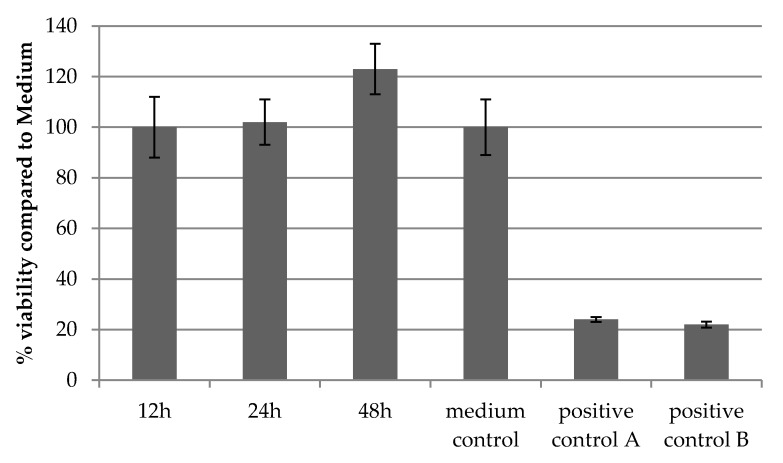
Confirmation of biocompatibility of PLA disks. MTT-tests were performed analogue to ISO 10993-5 and confirmed the biocompatibility of PLA disks as demonstrated by viability of cells after incubation for 12, 24 and 48 h in PLA medium, respectively, compared to standard cell medium. Medium control: standard cultivation medium; positive controls: ZDEC and ZDBC: polyurethane matrices stabilized with organic zinc.

In order to ensure biocompatibility of our PLA scaffolds, printed cages were incubated in medium for 12, 24 and 48 h. The supernatants were used for incubation of L929 cells seeded in 96 wells for 24 h and viability was tested with a MTT assay. This protocol is in accordance to ISO-10993-5: Biological evaluation of medical devices. Figure 3 demonstrates the viability of cells for all three time points. Cell viability of 100% corresponds to cells growing on tissue-culture-treated polystyrene substrates (medium control). According to ISO 10993-5, cell viabilities > 70% indicate no cytotoxic effects, whereas cell viability between 0% and 40% represents high cytotoxicity as seen in the positive controls A and B (ZDEC and ZDBC: polyurethane matrices stabilized with organic zinc) with values below 25% viability.

### 2.4. Microscopic Analyses

**Figure 4:**
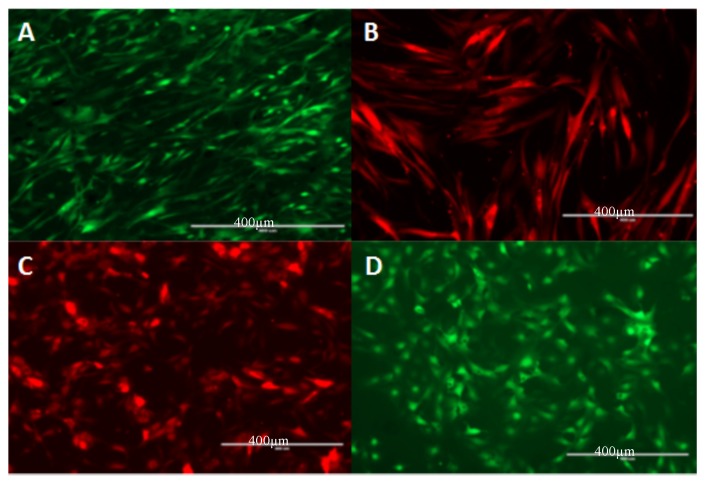
Microscopic analysis of different cell types seeded on PLA discs. (**A**) hOB (human primary osteoblasts; (**B**) normal human dermal fibroblasts: NHDF; (**C**) human umbilical vein endothelial cells: HUVEC; and (**D**) osteosarcoma cells: SaOS-2. Scale bars represent 400 µm.

Different cell types stably expressing eGFP (human primary osteoblasts: hOB, osteosarcoma cells: SaOS-2) or mCherry (human umbilical vein endothelial cells: HUVEC, normal human dermal fibroblasts: NHDF) were seeded on collagen-coated PLA discs to analyze cell growth. Figure 4 shows an equal distribution of all tested cells seeded on PLA discs.

### 2.5. Proliferation

**Figure 5:**
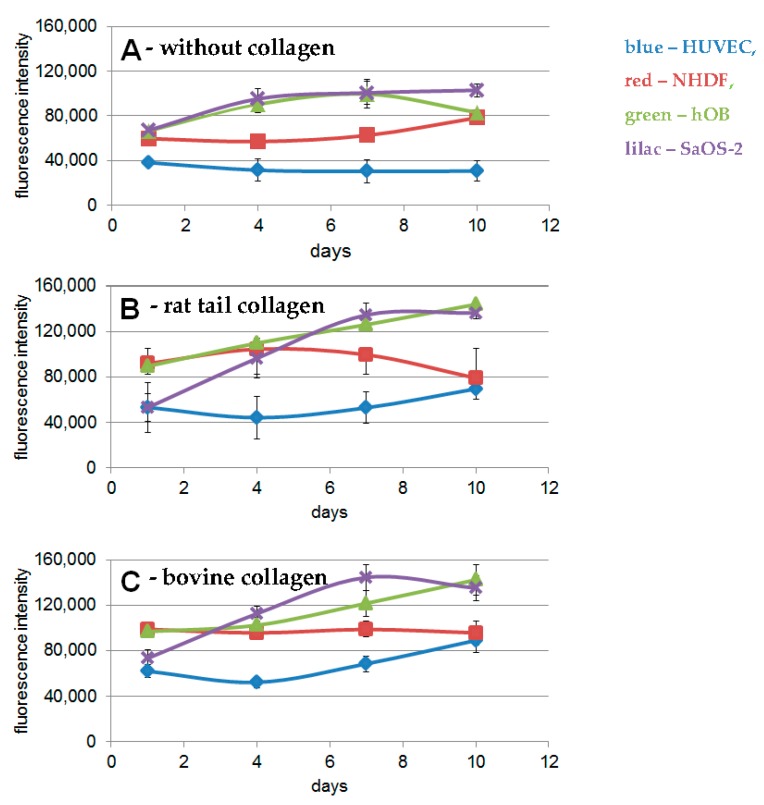
Proliferation assays on PLA discs with different cell types. (**A**) without collagen; (**B**) PLA discs coated with rat tail collagen; (**C**) PLA discs coated with bovine collagen. Blue: HUVEC, red: NHDF, green: hOB and lilac: SaOS-2.

For proliferation tests, we seeded cells on PLA scaffolds coated with or without collagen solutions. The results demonstrated statistically significant better proliferation of various cell types on PLA discs coated with rat tail or bovine collagen (Figure 5B,C), compared to non-coated discs (Figure 5A, *p* ≤ 0.05). The shape of these growth curves is similar, and, except for hOB cells the overall proliferation of the cells is slightly better on discs coated with bovine collagen, however, these differences are not statistically significant (*p* > 0.05).

It has been demonstrated that PLA is a biocompatible and biodegradable material [13,35,36]. However, only few in vitro studies have been performed concerning 3D-printed scaffolds employing PLA. One study was performed with murine MC3T3 cells, confirming the biocompatibility of PLA scaffolds. This study used different coatings (hyaluron and pullulan) and found different effects of the coatings on cells [30]. Rosenzweig et al. tested chondrocytes and nucleus pulposus cells on PLA scaffolds and demonstrated their increase in proliferation [37]. Yang et al. combined polylactide-co-glycolide (PGLA) with hydroxyapatite and found an osteoinductive effect with human bone marrow derived stem cells (hBMSCs) [38]. A similar study was performed by Senatov [39]. Wurm et al. manufactured PLA samples by fused deposition modeling and tested their biocompatibility with human fetal osteoblasts (hFOB) [40]. They demonstrated that their PLA samples showed no cytotoxicity, however, hFOB demonstrated reduced cell growth compared to polystyrene control, probably due to differences in surface roughness. These effects might be masked in our approach by collagen coating. Rodina tested migration and proliferation of murine mesenchymal stem cells on electrospun PLA scaffolds coated with collagen [41,42]—completely different to our scaffolds, but their study demonstrates possible combination of PLA and collagen. Concerning biocompatibility of PLA, our study is in accordance with the described literature, however, it is the first study analyzing 3D-printed PLA discs coated with collagen with different human cells in vitro. We could demonstrate that the cells adhere to collagen-coated PLA discs and exhibit their typical morphology. Moreover, all cell types proliferate on the discs over a period of ten days. This is especially interesting with regards to endothelial cells as these cells represent an angiogenic cell type and many studies showed that angiogenesis is the critical point of tissue regeneration in almost every tissue. Until now, only few groups demonstrated a positive effect on angiogenic factors of human stem cells in combination with polylactide [43,44,45].

### 2.6. SDF-1 Immobilization and Release

In order to construct a material which can be applied as bone substitute, a cage-like structure was printed with pores large enough for cells, vessels and/or bone to grow inside. To construct a material that enhances angiogenesis in the first place, we immobilized SDF-1 in the PLA collagen cage and measured the release kinetics of this factor from the cage.

**Figure 6:**
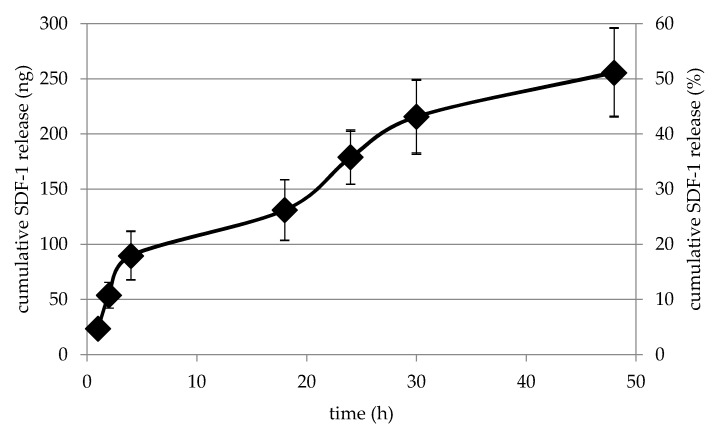
SDF-1 release from PLA collagen cages. SDF-1 is released from collagen inside the 3D-printed cages in a steady manner and after 48 h approximately 50% are still inside the cage.

For release kinetics, 500 ng SDF-1 were immobilized in collagen gel in PLA cages and its release was measured over 48 h. Interestingly, we could not observe a high initial burst release, but a relative steady release. After 48 h, 50% of the initially immobilized SDF-1 is still in the cage (Figure 6).

In former studies, we could demonstrated that SDF-1 bound to collagen or hydrogels keeps its functional bioactivity, induces angiogenetic effects and, as a consequence, supports bone tissue regeneration [46,47,48]. In these studies, we observed a high initial release of immobilized SDF-1, which was sufficient to induce angiogenetic effects; however, a steady release over a longer time period as observed in the present study with the PLA collagen cages seems to be preferable [11,49,50].

### 2.7. Angiogenic Potential

**Figure 7:**
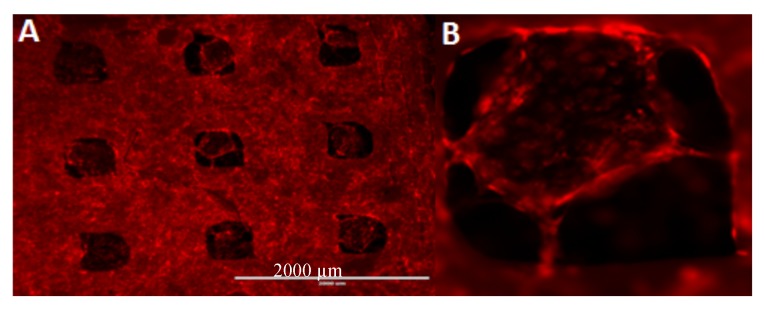
HUVECs seeded on PLA collagen–SDF-1 scaffolds. The cells adhere to the cage and seem to grow from the outside of the cage into the collagen gel (**A**). Thereby they start to form neo-vessel-like structures comparable to cells grown in Matrigel (**B**). Scale bar represents 2000 µm.

mCherry-expressing HUVECs were seeded on PLA cages loaded with SDF-1 immobilized in bovine collagen (Figure 7). The cells adhere to the cage and start to grow inside it. Thereby they start to form neo-vessel-like structures comparable to cells grown in Matrigel [51].

These results are promising concerning the application of mechanically stable PLA collagen–SDF-1 constructs as bone substitutes. The produced scaffolds are biocompatible, demonstrate a steady release of the immobilized factors and induce neo-vessel formation in endothelial cells. Until now, no such approach has been tested. Pinese et al. combined PLA polymers as knitted patches with collagen/chondroitin sulfate for ligament regeneration [52]. Heo et al. combined PLA with gelatin hydrogels and showed that human adipose-derived stem cells can be drifted to osteogenic differentiation and suggest this material for bone tissue engineering [53]. Yin used 3D-printed cages for cervical diseases [54] and Kao et al. coated PLA with poly(dopamine) for bone tissue engineering. They demonstrated a higher expression of ALP and osteocalcin in adipose-derived stem cells after seeding on these constructs. Moreover, they could show that some proteins associated with angiogenic differentiation were upregulated [43]. Concerning angiogenesis, Sekula et al. reported a positive effect of PLA on human umbilical cord-derived mesenchymal stem cells on gene expression of endothelial markers; however they did not analyze vessel formation in an angiogenesis assay [45]. Using an approach comparable to Kao [43], Yeh et al. demonstrated an upregulation of osteogenic and angiogenic markers of bone marrow stem cells on 3D-printed PLA scaffolds after immobilization of poly-dopamine [44]. To our knowledge, the present work is the only study testing angiogenic associated aspects with PLA scaffolds printed on a simple desktop 3D-printer coated or filled with collagen I employing inter alia endothelial cells and performing proliferation as well as angiogenesis assays.

Concerning in vivo evaluation of PLA constructs, Chou et al. used a PLA cage as a carrier for bone chips that induced bone regeneration in a rabbit model [13]. They demonstrated that morselized corticocancellous bone chips were converted into a structured cortical bone graft and that the PLA cage was already completely degraded 12 weeks after implantation. This demonstrates that the transfer of our in vitro results to in vivo studies is generally possible.

Our approach displays an inexpensive, easily constructed scaffold with the necessary mechanical stability and a soft material inside that can be modified with various cytokines or even cells to induce angiogenesis, and as a consequence bone regeneration. Proof of concept in a rat in vivo model is part of the follow-up-study. Before analyzing the potential of our scaffold to enhance bone regeneration in an in vivo femur defect model in the rat, further tests to characterize the mechanical stability of our 3D-prints loaded with collagen I will be performed. Mechanical tests as well as in vivo experiments will clarify the potential of our approach for bone tissue engineering.

## 3. Materials and Methods

### 3.1. 3D-Printing

For 3D-printing we used commercially available PLA filament (Ultimaker silver metallic PLA, iGo3D, Hannover, Germany) with a diameter of 2.85 ± 0.10 mm. Mechanical, thermal and other properties are listed in the technical data sheet from Ultimaker. Sanchez et al. give a good overview about material characterization methods for PLA filaments in 3D printing [55]. All PLA discs and cages were designed with a 3D modeling software (Autodesk^®^ Inventor Professional 2013, Autodesk, San Rafael, CA, USA) so that they fit to the 24 well of an ultra-low attachment plate (Corning, Wiesbaden, Germany). The 3D model file generated by the Autodesk Inventor software was exported to a convenient file format (STL).

For the 3D print pre-processing this file was imported into the CURA 2.5 software (Ultimaker B.V., Geldermasen, The Netherlands), previewed, scaled and adjusted as necessary. Cura slices the model ready for print with the 3D-printer Ultimaker 2+ (Ultimaker B.V., Geldermasen, The Netherlands) employing the smallest available nozzle size (0.25 mm). A slightly modified high quality profile in Cura 2.5 with a layer height of 0.06 mm, 100% infill, 200 °C nozzle temperature and 60 °C build plate temperature has shown the best results.

**Figure 8:**
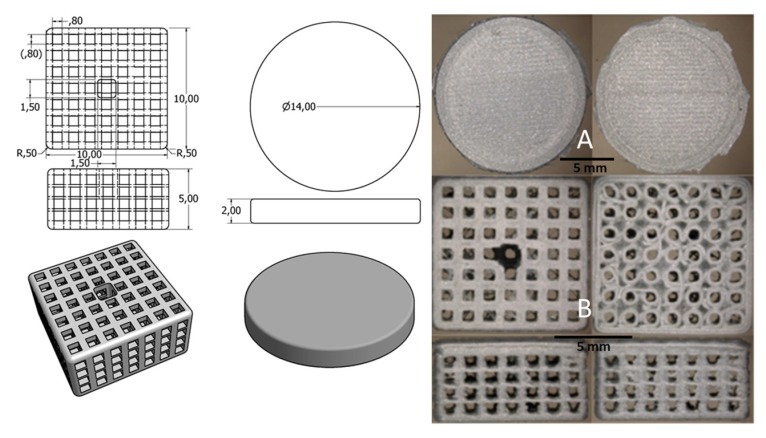
**Left side**: Technical sketches with dimensions in [mm] for both the PLA disc and the PLA cage; **Right side**: light microscopic pictures of the original 3D-printed objects (**A**) PLA disc; (**B**) PLA cage.

Figure 8 demonstrates the difference between the designed 3D model and the 3D printed objects.

### 3.2. Quality Control

For quality control purposes, all the printed objects underwent detailed examination by means of stereo light microcopy (Leica MZ 16A) as mentioned above, scanning electron microscopy (FEI Quanta 200FEG) and micro computational tomography (Scanco µCT40). SEM investigation was done under low vacuum condition to avoid disturbing artefacts due to the electrical isolating characteristic of the PLA material. The SEM investigation under low vacuum condition is recommended especially for non-conductive samples with undergoing cavities were the available sputter coating techniques fails to cover the whole sample surfaces.

**Figure 9:**
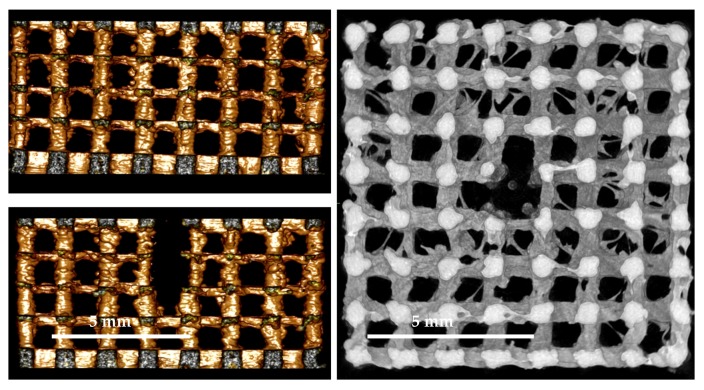
SEM quality control. Magnification: 100×.

**Figure 10:**
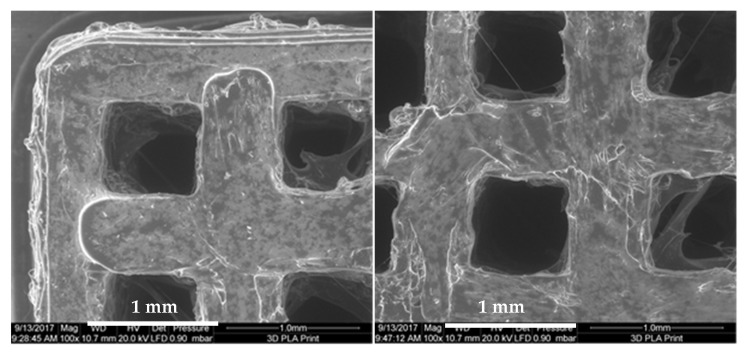
µCT quality control. (**Left**): surface rendered images (view from both sides); (**Right**): MPR image with volume rendering from the internal site of the PLA cage.

Parts of the PLA cage surfaces are shown in Figure 9 to demonstrate the limits of precision of the applied 3D printing technique. To make sure that a high degree of connectivity was achieved for the printed material the PLA cages were scanned with a micro-CT system. Micro-CT reconstructions as shown in Figure 10 demonstrate the internal designed structures with a sufficient porosity.

### 3.3. Biocompatibility

In vitro cytotoxicity was analyzed employing the MTT ([3-(4,5-dimethylthiazol-2-yl)-2,5-diphenyltetrazoliumbromid)]) assay analogous to ISO 10993-5. Mouse L929 cells (20.000 cells/well) were seeded in a 96-well tissue culture polystyrene (TCPS) plate for 24 h. Directly after printing PLA constructs were incubated in 500 µL cell media for 24 h. 100 µL of this extract were given to L929 cells in the 96 well plate. After an incubation time of 24 h the MTT assay was performed according to ISO 10993-5. The colorimetric readout was performed at a wavelength of 570 nm (reference wavelength 650 nm). Polyurethane membranes stabilized with organic zinc derivatives ZDEC (zinc diethyl dithiocarbamate) and ZDBC (zinc dibutyl dithiocarbamate) (Food and Drug Safety Center, Hatano Research Institute, Hadano, Japan), were used as positive controls. These controls induce a reproducible cytotoxic reaction.

### 3.4. Endotoxin Contamination

Prior to the cell experiments, the 3D-printed PLA constructs were tested for endotoxin contamination employing the endpoint chromogenic LAL (limulus amebocyte lysate) assay (Lonza, Basel, Switzerland). The assay was performed according to the manufacturer’s recommendations. For preparation of the endotoxin analyte solution, PLA discs and cages were incubated in 1 mL water directly after printing without preceding washing-steps for 24 h at 37 °C (conditions were transferred from ISO 10993-5: 2009; Biological evaluation of medical devices) in order to extract any potential endotoxin from the PLA matrix. the supernatant (50 µL) was employed in the LAL test. Parallel to sampling of the analyte solutions, a standard curve was established and the analysis results compared to positive controls (provided by the manufacturer (Lonza, Basel, Switzerland) within the LAL kit) and negative control (pure endotoxin free water).

### 3.5. Coating/Filling of Discs/Cages with Collagen I

Solid PLA discs were coated with bovine (Viscofan, Weinheim, Germany) or rat tail collagen (Invitrogen, Karlsruhe, Germany). Collagens were diluted 1:100 with PBS and discs were incubated for one hour to assure even coating.

3D cages were filled with a collagen gel solution following an established protocol [56,57]. Briefly, collagen type I (3 mg/mL bovine, Viscofan, Weinheim, Germany), aqua dest, M199 (10×), NaHCO_3_ and NaOH and SDF-1 (500 ng, Miltenyi, Bergisch Gladbach, Germany) were combined within an ice bath to prevent polymerization of the solution. Next, 300 μL of the liquid collagen solution was pipetted into a PLA cage sitting in a 24-well ultra-low attachment plate (Corning, Wiesbaden, Germany) and allowed to polymerize.

### 3.6. Cells

Four cell types were used to analyze cell growth and viability on 3D-printed PLA discs coated with bovine or rat tail collagen. Normal human dermal fibroblasts (NHDF, Promega, Karlsruhe, Germany), human primary osteoblasts (hOB [48,58]), osteoblast-like cells (SaOS, ATCC, Manassas, VA, USA) and endothelial cells (HUVEC, Promega, Karlsruhe, Germany) were seeded onto discs, into or onto cages, respectively, sitting in a 24-well ultra-low attachment plate. Cells (100,000) were seeded in all cases.

### 3.7. Viability

Proliferation was measured over a 10-day period with alamarBlue-assay (Invitrogen, Karlsruhe, Germany) according to the manufacturer’s protocol.

To allow analysis by fluorescent microscopy cells were transduced with lentiviral vectors encoding mCherry or enhanced green fluorescent protein. Vector supernatants were collected and concentrated from transfected 293T producer cells as previously described [59]. For gene transfer, 15.000 human osteoblasts (hOB) or HUVEC were seeded into 24-well tissue culture plates (Greiner, Frickenhausen, Germany) in 500 µL media supplemented with 5 µg/mL protamine sulfate. Two rounds of transduction on day 1 and 3 were performed at a cumulative multiplicity of infection (MOI) of ~100 to achieve >98% gene marking. Transduction efficiency was confirmed by fluorescence microscopy (Wilovert AFL30, Hundt, Wetzlar, Germany) and flow cytometry FACSCalibur (BD Biosciences, San Jose, CA, USA) using the CellQuestPro Software (BD Biosciences, San Jose, CA, USA). The different cell types were seeded as monocultures on PLA discs or on the cages and the spreading, morphology and distribution of the cells analyzed microscopically with a fluorescent microscope.

### 3.8. SDF-1 Release Assay

Three-dimensional cages were filled with bovine or rat tail collagen immobilized with fluorescein-linked SDF-1. Release kinetics of SDF-1-FITC from the PLA collagen–cages were measured via fluorescence reading in the supernatant (Glomax-Multidetection System, Promega, Karlsruhe, Germany).

### 3.9. Statistical Analysis

Statistical analysis was performed using SPSS 22.0 software (SPSS Inc., Chicago, IL, USA). Results are presented as means ± standard deviation. At least triplicate measurements for each time point and experimental condition were performed. Differences corresponding to *p* < 0.05 were considered statistically significant.

## 4. Conclusions

Polylactide is an interesting material for 3D-printing in biomaterial research. This study confirms its biocompatibility and demonstrates an endotoxin contamination clearly below the FDA limit. Cells of various cell types (osteoblasts, fibroblasts and endothelial cells) grow, spread and proliferate on PLA-printed discs. PLA cages loaded with SDF-1–collagen support cell growth of endothelial cells and induce neo-vessel formation. These results demonstrate the potential for PLA scaffolds in medical applications, for example, in bone tissue engineering. Tests of mechanical stability as well as in vivo tests employing a femur defect model in the rat will define the potential to induce angiogenesis and bone regeneration of the described scaffolds.
